# Protective Roles of Folic Acid in the Responses of Bovine Mammary Epithelial Cells to Different Virulent *Staphylococcus aureus* Strains

**DOI:** 10.3390/biology10111164

**Published:** 2021-11-12

**Authors:** Siyuan Mi, Yongjie Tang, Liangyu Shi, Xueqin Liu, Jingfang Si, Yuelin Yao, Serafino M. A. Augustino, Lingzhao Fang, Ying Yu

**Affiliations:** 1 National Engineering Laboratory for Animal Breeding and Key Laboratory of Animal Genetics, Breeding, and Reproduction, Ministry of Agriculture, College of Animal Science and Technology, China Agricultural University, Beijing 100193, China; caumsy@cau.edu.cn (S.M.); cautyj@163.com (Y.T.); shiliangyu@cau.edu.cn (L.S.); cauliuxueqin@163.com (X.L.); sijingfang@cau.edu.cn (J.S.); serafino156@gmail.com (S.M.A.A.); Lingzhao.fang@igmm.ed.ac.uk (L.F.); 2 MRC Human Genetics Unit, Institute of Genetics and Molecular Medicine, University of Edinburgh, Edinburgh EH4 2XU, UK; Y.Yao-38@sms.ed.ac.uk; 3 College of Natural Resources and Environmental Studies, University of Juba, Juba P.O. Box 82, South Sudan

**Keywords:** bovine mammary epithelial cells, *Staphylococcus aureus*, folic acid, host and pathogen interactions, RNA-seq, DNA sensing pathway

## Abstract

**Simple Summary:**

Mastitis caused by zoonotic *Staphylococcus aureus* (*S. aureus*) is hard to cure. The cross-talk between host, pathogen, and environment influences bovine resistance to *S. aureus* infection. Our previous study observed that the inflammation of bovine mammary gland could be reduced by folic acid, i.e., a kind of beneficial micronutrient. However, the heterogeneity of inflammatory responses in bovine mammary epithelial cells challenged with different virulent *S. aureus* strains and the protective effects of folic acid on improving host defense against *S. aureus* infection are still unclear. RNA-seq was performed to investigate the effects of different *S. aureus* lineages and folic acid on the global transcriptome of bovine mammary epithelial cells (Mac-T cell line). Functional enrichment analysis indicated different *S. aureus* strains share few differentially expressed and spliced genes and activate host differentially inflammatory responses, and folic acid plays the protective role in host defense against the *S. aureus* challenge partially through activating cytoplasmic DNA sensing and tight junction pathway. This study provides novel insights into the improvement of *S. aureus* mastitis defense.

**Abstract:**

Mastitis caused by *Staphylococcus aureus* (*S. aureus*) infection is one of the most difficult diseases to treat in dairy cattle. Exploring the biological progression of *S. aureus* mastitis via the interaction between host, pathogen, and environment is the key to an effective and sustainable improvement of animal health. Here, two strains of *S. aureus* and a strain of MRSA (Methicillin-resistant *Staphylococcus aureus*) isolated from cows with different inflammation phenotypes were used to challenge Mac-T cells and to investigate their effects on the global transcriptome of the cells, then to explore the potential regulatory mechanisms of folic acid on *S. aureus* mastitis prevention. Differential gene expression or splicing analysis showed that different strains of *S. aureus* led to distinct transcriptional responses from the host immune system. Folic acid could protect host defense against the challenge of *S. aureus* and MRSA partially through activating cytoplasmic DNA sensing and tight junction pathway. *ZBP1* at the upstream of cytoplasmic DNA sensing pathway was verified and related to anti-pathogen through RNA interference. Further enrichment analysis using these transcriptome data with cattle large-scale genome-wide association study (GWAS) data confirmed that *ZBP1* gene is highly associated with bovine somatic cell score (SCS) trait. Our data shed light on the potential effect of FA through regulating key gene and then protect host cells’ defense against *S. aureus* and MRSA.

## 1. Introduction

*Staphylococcus aureus* (*S. aureus*) is a zoonotic bacterium that is widely spread in various environments, including human and animal skin, nose, throat, stomach, carbuncle, air, and sewage. It is an important cause of bovine mastitis and a threat to human health [1]. Owing to its immune evasion and resistance to multiple antibiotics, *S. aureus* mastitis is hard to cure [2,3,4]. The improper clinical use of antibiotics has resulted in the appearance of methicillin-resistant *S. aureus* (MRSA) [5,6,7]. Thus, understanding the progression of bovine *S. aureus* mastitis is vital for its prevention.

Prevention strategies for pathogen-induced diseases need to be developed from the perspectives of both pathogens and host cells. In the mastitis cell model, a previous study observed that the transcriptional expression levels of several candidate genes in primary bovine mammary epithelial cells (pbMECs) or Mac-T cells line are significantly different for different *S. aureus* lineages challenge [8]. For the host, the mammary epithelial cells in mammary gland establishes the first physical line of defense against the external pathogen, and a healthy mammary gland is vital in mobilizing the early innate immune response [9,10]. In our previous study, we isolated and cultured 191 *S. aureus* strains, including five MRSA strains, from bovine milk across nine Holstein dairy farms [11,12], and found that *S. aureus* is widely distributed in cows with different inflammatory responses. In addition to the *S. aureus* isolated from mastitis cows, some *S. aureus* were also isolated from cows with asymptomatic phenotype. The asymptomatic cases are usually not detected and then furtively spreads throughout the herd, posing a huge threat for cows. The degree of mammary inflammation depends on the pathogen, the host immunity level, and their interaction. Therefore, the similarities and differences of host responses to different *S. aureus* strains need to be investigated.

In addition, certain micronutrients may be beneficial and environmentally sound for supporting host immune function [13,14,15]. Folic acid (FA) plays a crucial role in the development of mammary gland [14], and reduces certain inflammation in some species (such as cattle [16], chicken [17], and mice [18,19]), as well as cancer risks in mice [18] and human [20]. FA supplementation reduces postpartum SCC during the periparturient period and improves production traits in dairy cattle [21,22]. However, the effect of FA on mammary epithelial cells during the challenge of different *S. aureus* strains has not been systematically studied.

In this work, three distinct strains of *S. aureus* and FA were used to treat Mac-T cells, and then the characteristics of transcriptomic responses were investigated. Bioinformatics analysis and experimental validation revealed that FA might act as an inflammatory regulator by influencing the expression of *ZBP1* and genes within the tight junction pathway. The results describe the cross-talk between *S. aureus,* FA, and Mac-T, and provide novel insights into the prevention of zoonotic pathogen infection in livestock.

## 2. Materials and Methods

All the experiments in this study were approved by the Animal Welfare Committee of China Agricultural University, Beijing, China.

### 2.1. Bacterial Strains

Twenty-one re-sequenced *S. aureus* strains (Figure S1) were randomly selected from 191 *S. aureus* strains, which were originally isolated from milk samples of 1112 lactating Holstein cows [11]. Among 21 strains of *S. aureus*, three strains were finally selected for this study. Briefly, the first strain of *S. aureus* was isolated from the cow with low SCC (<100,000/mL, named as L strain) and without mastitis symptoms. The second one was isolated from the cow presenting with clinical mastitis (named as M strain). The third strain is MRSA and was isolated from a clinical mastitis cow (named as MM strain). The three strains were re-identified by *nuc*- and *mecA*-specific polymerase chain reaction (PCR), as described in our previous study [12].

Afterward, 50 μL of bacteria solution was inoculated into 5 mL of sterile tryptone liquid medium and incubated at 37 °C at 200 rpm/min for 18–24 h. Plate count agar (PCA) was used for bacterial counting. According to the number of colonies, the concentration of bacterial solution was calculated and adjusted to 1 × 10^8^ CFU/mL, and the solution was stored at 4 °C for later use.

### 2.2. DNA Extraction, Whole Sequencing and Biofilm Assay of S. aureus Strains

Genome DNA of *S. aureus* strains were extracted using the bacterial genomic DNA extraction kit (TIANGEN, Beijing, China), according to the manufacturer’s instructions. The quality and concentration of DNA was checked on 1% agarose gels and NanoDrop 2000, respectively. Then, qualified DNA was provided for library construction. Furthermore, a Illumina HiSeq2500 machine was used for sequencing after library construction. Then, NGS QC Toolkit v2.3.3 software was used for data quality control, and SPAdes v3.13.0 software was applied to assemble the reads. The assembled contigs were uploaded to CGE Server (https://cge.cbs.dtu.dk/services/MLST/, accessed on 15 January 2018) for *S. aureus* multilocus sequence typing (MLST), and appropriate reference genomes were selected according to the MLST results. The reads of the original sequence assembled into contigs were analyzed according to the reference genomes. Mauve v2.3.0 was used to sort the contigs by selecting “Move Contigs” in the menu using default parameters. After iteration, the whole genome sequence could be obtained by sequencing according to the reference genome. *S. aureus* whole genome sequence statistics are shown in Table S1. A phylogenetic tree was constructed for the 21 re-sequenced *S. aureus* strains and 4 reference *S. aureus* genomes (*ATCC_6538*, *MW2*, *GD1677,* and *BA01611*) (Figure S1).

A bacterial biofilm assay was performed for the selected three *S. aureus* strains using crystal violet, according to a previous study [23]. The optical density (OD) value of the solution in the culture wells for each sample was determined by a SpectraMax i3x Multi-Mode Microplate Reader (Molecular Devices, San Jose, CA, USA) at 590 nm. The culture medium of uninoculated bacteria was used as the negative control.

### 2.3. Cell Culture, S. aureus Challenge and Folic Acid Treatment of Mac-T Cells

After the cell culture of continuous three generations (48 h per generation), stable Mac-T cells were re-suspended in complete culture medium and inoculated in a six-well plate with an inoculation amount of 1 × 10^6^ cells/well. The medium was discarded when the logarithmic stage was reached. Dulbecco’s modified Eagle’s medium (DMEM) was added and cells were incubated at 37 °C for 2 h. The previous DMEM culture medium was discarded, and the cells were incubated with DMEM culture medium containing 5 μg/mL FA (prepared in advance with folic acid powder, Sigma-Aldrich, St. Louis, MO, USA) for 24 h, and a corresponding control group without FA was set. The culture medium was discarded and cells were challenged with 1 × 10^8^ CFU/mL *S. aureus* solution for 6 h, with the multiplicity of infection (MOI) of bacteria to cell set to 10:1. After *S. aureus* was challenged, the six-well plate was washed with sterile PBS for three times. Finally, 1 mL of Trizol was added to lyse the cells from the plate, and the specimen was stored at −80 °C freezer for later use.

**Figure 1:**
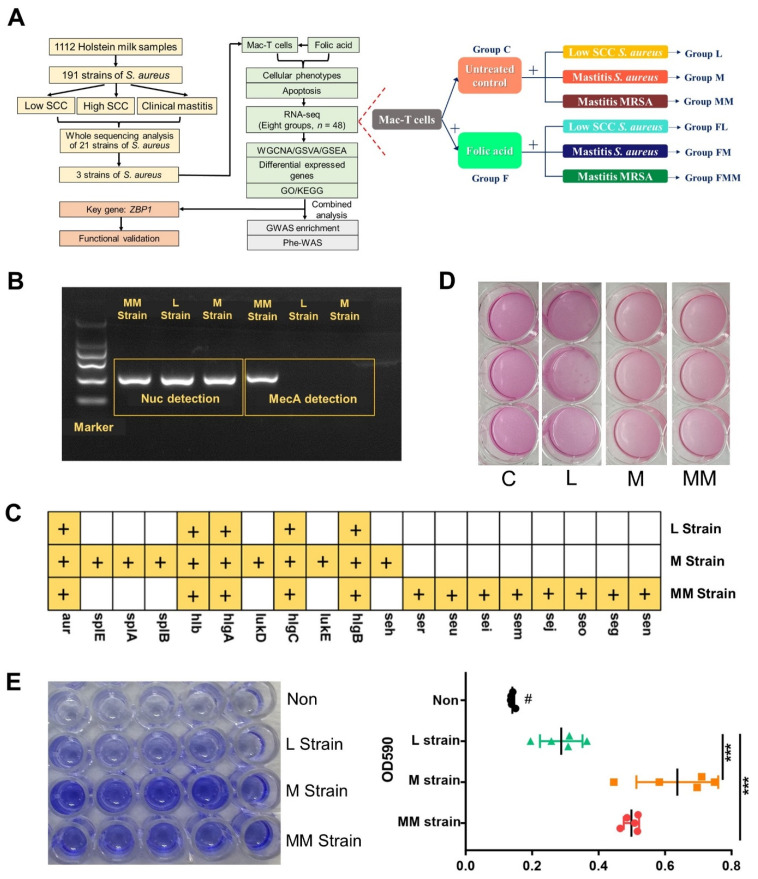
Experimental design and characteristics of three *S. aureus* strains. (**A**) Overview of experimental design. Eight groups of Mac-T cells include Group C: untreated control; Group L: treated with L strain of *S. aureus*; Group M: treated with M strain of *S. aureus*; Group MM: treated with MM strain of MRSA; Group F: treated with folic acid; Group FL: folic acid treatment plus L treatment; Group FM: folic acid treatment plus M treatment; Group FMM: folic acid treatment plus MM treatment. The colors for the groups are the same in all figures. (**B**) Detection of *S. aureus*/MRSA by *nuc*- and *mecA*-specific PCR. L strain: *S. aureus* strain from cattle with low SCC; M strain: *S. aureus* strain from mastitis cattle; MM strain: MRSA strain from mastitis cattle. (**C**) Virulence factors genes of the three *S. aureus* strains identified by re-sequencing. (**D**) Cytotoxicity of these three *S. aureus* strains to cells. Darker red media indicates a low number of dead cells, while lighter red media indicates high number of dead cells. The MOI is 50:1. (**E**) Biofilm-forming ability of the three *S. aureus* strains tested by crystal violet solution. In the left panel, darker blue media indicates strong biofilm forming ability, while lighter blue media indicates weak biofilm formation ability. “Non” represents no bacteria added to the negative group. In the right panel, the OD value at 590 nm for different groups (*** *p* < 0.001). “#” means that the *p* < 0.001 compared with the other 3 groups.

In total, eight Mac-T cells groups were designed based on the challenge of *S. aureus* and FA treatment (right panel in Figure 1A): C is the untreated control group; L is treated by an L strain of *S. aureus*; M is treated by a M strain of *S. aureus*; MM is treated by an MM strain of MRSA; F is the folic acid-treated group; FL is treated by folic acid before challenged with L strain of *S. aureus*; FM is treated by folic acid before challenged with M strain of *S. aureus*; and FMM is treated by folic acid before challenged with MM strain of MRSA.

### 2.4. RNA Extraction and RNA-Seq

Trizol Reagent (Invitrogen) was used to extract total RNA from the treated Mac-T cells. After the integrity number (RIN) of 48 RNA samples was qualified, cDNA library was constructed, and then sequencing was completed by an Illumina HiSeq2500 machine (Novegene Co., Ltd., Beijing, China). Finally, paired end reads at 150 bp in length were obtained. FastQC v0.11.8 software was used to evaluate the quality of the clean data obtained from sequencing, and NGSQCToolkit v2.3.3 was applied to control and filter the reads.

### 2.5. RNA-Seq Data Analysis

After the quality control of raw RNA-seq data, Hisat2 v2.1.0 software was employed for genome assembly, and the resulting SAM files were converted to BAM files by SAMtools v1.9. FeatureCounts (subread package v1.6.3) software was utilized to generate the read counts for each sample. The genes expressed in at least one sample were retained, and the corrected read counts were obtained by DEseq2 v1.28.1.

Gene co-expression network analysis was conducted by weighted gene correlation network analysis (WGCNA, v1.70-3). The corrected read counts larger than zero in all sample were employed for WGCNA analysis. Eight different treatments for the eight groups were regarded as eight phenotypic traits. The soft threshold power was set to 10 according to the analysis results, and the adjacency matrix was constructed. With the dynamic hybrid cutting method, 17 co-expression modules were clustered. The minimum module size was set to 100 and the merge cut height was set to 0.05. KEGG analysis was performed on all 17 modules, and KEGG pathways in important modules were identified.

For the eight experimental groups, gene set variation analysis (GSVA, v1.36.3) was then employed to analyze the hallmark gene sets which contain 50 characteristic gene sets in the MSigDB database for all samples. In addition, gene set enrichment analysis (GSEA, v4.0.3) was performed.

Next, differentially expressed genes of *q* < 0.05 and log_2_|Fold change| > 1 were chosen for further analysis. The detailed mapping information of RNA-seq analysis for all samples is shown in Table S1. With regard to alternative splicing analysis, STAR v2.7.5 software was used for genome assembly to obtain BAM files. Code built-in Leafcutter v0.2.9 was employed to convert BAM into junc files. Leafcutter default parameters were used for the differential splicing analysis of pair-wise comparison. Clusters with a *q* value of <0.05 were chosen for further analysis.

The number of differentially expressed genes (DEGs) and differentially spliced genes (DSGs) in various comparison sets was shown by UpSetR v1.4.0. For the GO and KEGG enrichment analysis, a web-based gene set analysis toolkit (WebGestalt, http://www.webgestalt.org/, accessed on 6 December 2020) was used. The GO terms and KEGG pathways with enrichment ranking top 10 based on the *p* value were obtained. For hub gene analysis, the gene set was imported into STRING (https://string-db.org/, accessed on 28 August 2020), and the TSV file of interaction among these genes was obtained. CytoHubba within Cytoscape v3.7.2 software was then used for hub gene analysis, and DMNC parameters were used as the rank criteria. I-TASSER software (https://zhanglab.ccmb.med.umich.edu/I-TASSER/, accessed on 11 November 2020) was used for protein structure and function predictions [24]. VMD v1.9.4 was used for the visualization of protein structure.

### 2.6. RNA Interference

A total of 5 × 10^5^/mL cells were inoculated into six-well plates and added with 2 mL of complete medium to each well. When the cell confluence reached 50–80%, the complete medium was discarded. Then, 2 mL of basic DMEM supplemented with 100 pmol of siRNA and 5 μL of transfection reagent lipofectamine 2000 (Thermo Fisher Scientific, Waltham, MA, USA) was added to each well. After 6 h of transfection, the complete medium was discarded, and PBS was used to wash the cells twice. Next, cells were cultured in DMEM complete medium for 40 h. The primers for *ZBP1* gene interference fragment are as follows: forward (5′-3′) GCGAGUUCAGAUGGGACAUTT reverse (5′-3′) AUGUCCCAUCUGAACUCGCTT.

### 2.7. PCR and Fluorescence Quantitative PCR

Extracted bacterial genomic DNA was used as the PCR template, and PCR was conducted according to the *mecA* and *nuc* sequences of specific genes of MRSA and *S. aureus*. The *mecA*- and *nuc*-specific primers are shown in the Table 1. The primer sequences were synthesized by TSINGKE Biotechnology Co., Ltd. (Beijing, China). The PCR system is composed of DNA template, primer, and PCR master mix (TSINGKE Biotechnology, Beijing, China). 

The LightCycler^®^ 480 SYBR Green I Master kit (Roche, Basel, Switzerland) was used for fluorescence quantitative PCR. The bovine gene *GAPDH* was used as the reference gene. Two technical replicates were prepared for each cDNA sample, and the mean Ct value was used to calculate the genes relative expression by 2^−ΔΔCt^ method. The significant differences between groups were examined by a Student’s t-test.

### 2.8. Cell Cytotoxicity-Related Assays

A total of 10^5^ cells were inoculated into 96-well plates and added with 0.1 mL of complete medium to each well. When the logarithmic stage was reached, cells were treated with DMEM culture medium containing different concentration of FA (0.001, 0.005, 0.01, 0.025, 0.05, 0.1, 0.25, 0.5, 1, 5, 10, and 20 μg/mL) for 24 h, then cells were challenged with *S. aureus* solution for 6 h, with the MOI set to 10:1. After that, an LDH cytotoxicity assay kit (Beyotime, Shanghai, China) was used to determine cell cytotoxicity. All the determination steps were performed following the manufacturer’s instructions. Finally, cell samples were visualized under a SpectraMax i3x Multi-Mode Microplate Reader (Molecular Devices, San Jose, CA, USA).

A total of 2.5 × 10^5^ cells were inoculated into 24-well plates and added with 0.5 mL of complete medium to each well. When the logarithmic stage was reached, cells were treated with DMEM culture medium containing 5 μg/mL of FA for 24 h. Then, cells were challenged with *S. aureus* solution for 6 h, with the MOI set to 10:1. After that, mitochondrial membrane potential and an apoptosis detection kit (Beyotime, Shanghai, China) was used to determine cell apoptosis. All the determination steps were performed following the manufacturer’s instructions. Finally, cell samples were visualized under a Nikon ECLIPSE Ts2 fluorescence microscope (Nikon, Tokyo, Japan).

A total of 2.5 × 10^5^ cells were inoculated into 24-well plates and added with 0.5 mL of complete medium to each well. When the logarithmic stage was reached, RNA interference for *ZBP1* gene was conducted. Then, cells were challenged with *S. aureus* strain Newman solution for 6 h, with the MOI set to 10:1. After that, apoptosis and necrosis detection kit (Beyotime, Shanghai, China) was used to determine cell apoptosis and necrosis. All the determination steps were performed following the manufacturer’s instructions. Finally, cell samples were visualized under a Nikon ECLIPSE Ts2 fluorescence microscope (Nikon, Tokyo, Japan). The whole process was carried out in the dark.

### 2.9. GWAS Enrichment Analysis and Phenome-Wide Association Analysis (Phe-WAS)

Cattle GWAS data (*n* = 27, 214) is from https://figshare.com/s/ea726fa95a5bac158ac1 [25] and https://figshare.com/s/94540148512dddf7ed32 (accessed on 29 October 2020) [26], and 23 complex traits related to production, reproduction, and health were chosen for this study. The candidate gene set consists of 5 modules from WGCNA, 16 gene sets from DEGs, and 15 gene sets from DSGs. GWAS enrichment analysis of these candidate gene sets were performed based on count-based approach [27], and *p* < 0.05 was set as the significant cut-off.

Afterwards, several key candidate genes from above analysis were selected, and the upstream and downstream of these genes ± 10kb were searched for significant SCS GWAS signals (*p* < 1 × 10^−5^). Intersect function of BEDTools was used to overlap SNP and its located or nearby genes. These key candidate genes of cattle were used for phenome-wide association study (Phe-WAS) to explore the function of these homologous genes in human complex traits (https://atlas.ctglab.nl/, accessed on 22 December 2020) [28].

## 3. Results

### 3.1. Phenotypic Discrepancies of Three Bovine-Originated S. aureus Strains

A total of 21 strains of *S. aureus* isolated from the milk of 21 Holstein cows were re-sequenced on genome-wide (Figure S1). Each strain obtained around 1 Gb of data. Three different *S. aureus* strains were selected for the current study. Two strains of *S. aureus* and one strain of MRSA were reconfirmed by *nuc*- and *mecA*-specific PCR before challenge, respectively (Figure 1B). The first and the second strains of *S. aureus* were isolated from the milk of cows with low SCC (L strain, <100,000/mL) and that with mastitis symptoms (M strain), respectively. The strain of MRSA was isolated from milk of cow with mastitis symptoms (MM strain).

We observed that M and MM strains had more virulence genes (Figure 1C) and a higher toxicity to Mac-T with more death cells in vitro compared with strain L at MOI 50 (Figure 1D). The bacterial biofilm was assessed separately to further distinguish differences among the three *S. aureus* strains. M and MM strains showed greater forming ability of biofilm than L strain (Figure 1E). These features of these *S. aureus* strains correspond to the phenotypic observations of mastitis in cows.

### 3.2. Transcriptomic Features of Host Cellular Responses to Three S. aureus Strains Challenged with or without FA Supplementation

Eight groups corresponding eight treatments were set for transcriptome analyses using Mac-T cells (6 replicates per treatment, *n* = 48; right panel in Figure 1A). Transcriptomes among the eight groups were compared using three approaches: (1) WGCNA was used to get the set of co-expressed genes in each group; (2) GSEA and GSVA were conducted to understand the biological functions relevant with transcriptional alterations induced by *S. aureus* and FA; and (3) differentially expressed or spliced genes were detected.

**Figure 2:**
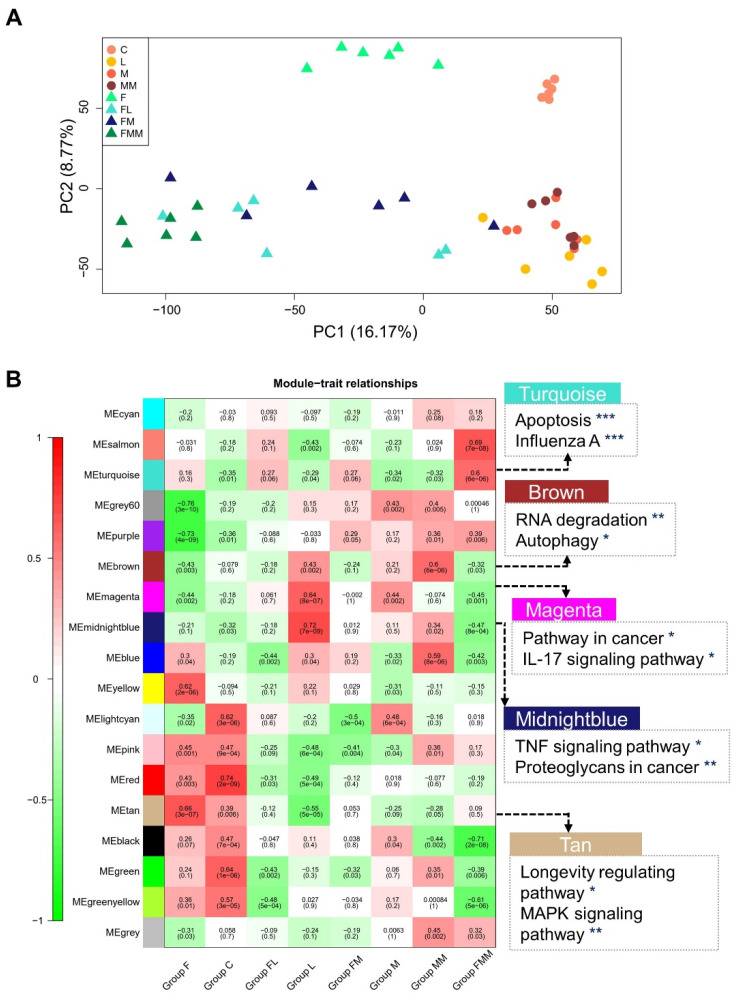
Five key WGCNA modules related to important KEGG pathways of Mac-T cells post FA treatment and bacteria challenge. (**A**) Principal component analysis (PCA) of 48 samples. (**B**) WGCNA analysis reveals the relationships between each module in each row and each treatment in each column. And the value outside the bracket represents the correlation coefficient, the value in the bracket is the *p* value. (* *p* < 0.05, ** *p* < 0.01 and *** *p* < 0.001). Five key WGCNA modules and partially enriched KEGG pathways are listed on the right side.

No significant difference was observed in the distribution of gene expression levels among the eight groups (Figure S2A), and there was high consistency within each group (Figure S2B,C,E). In addition, principal component analysis (PCA) and hierarchical clustering of all 48 samples illustrated that FA supplementation was one of the main influencing factors of samples difference (Figure 2A), which can classify the groups as left side and right side. The other main factor is *S. aureus* challenge, which can classify the groups as upper side and lower side (Figure 2A).

### 3.3. Key Gene Co-Expression Modules and Gene Sets Enriched in Immune-Related Pathways

WGCNA was conducted to detect the gene co-expression modules and their relationships among the eight groups. A total of 13,629 expressed genes were identified and divided into 18 modules, of which 17 were co-expression modules and 1 module without cooperative expression behavior (Figure 2B). The number of genes in each module ranged from 154 to 3681 (Table S2). The genes in the modules that are significantly (*p* < 0.05) associated with the treatments of eight groups were selected for KEGG pathway enrichment (Figure 2B). The modules, i.e., magenta, turquoise, and midnight blue, showed an enrichment of genes related to inflammation or cancer processes, such as apoptosis, IL-17 signaling pathway, and proteoglycans in cancer. In addition, these modules are significantly (*p* < 0.01) related with FA treatment or *S. aureus* (MRSA) challenge (Figure 2B). The tan module was enriched in longevity regulating pathway, positively related with unchallenged groups (groups C and F), and negatively associated with *S. aureus*-challenged groups (groups L, M, and MM). These findings imply that *S. aureus* may influence the longevity of bovine mammary epithelial cells by regulating these genes within the tan module. Moreover, the brown module was enriched in RNA degradation, was negatively related with FA treatment, and was positively associated with *S. aureus* treatment. This finding was consistent with DEGs results that after FA treatment, 69.83–78.84% DEGs are up-regulated (Table S3).

**Figure 3:**
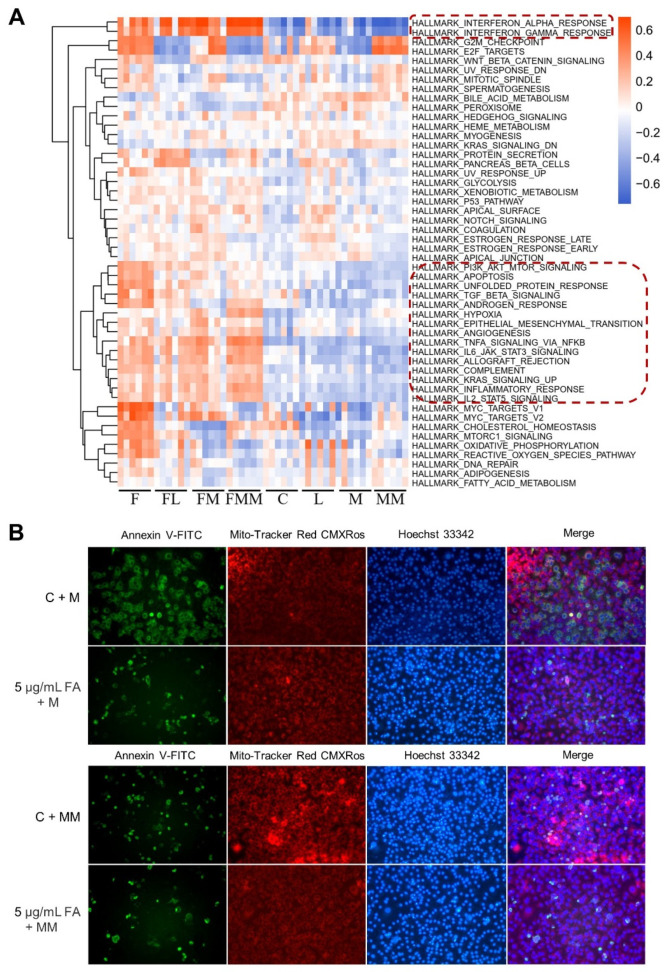
The immune response and apoptosis detection of Mac-T cells after FA treatment and/or *S. aureus* challenge. (**A**) Heatmap of eight groups in hallmark gene sets by GSVA analysis. The dotted red boxes highlight the immunity-related gene sets. (**B**) FA of 5 μg/mL can reduce the apoptosis induced by *S. aureus* challenge in mitochondrial membrane potential and apoptosis detection. Apoptotic cells were strained green by Annexin V-FITC; Cells were strained red by Mito-Tracker Red CMXRos; Cell nucleus were strained blue by Hoechst 33342.

GSEA and GSVA were performed to investigate the characteristics of hallmark gene sets in each group, and differentially expressed genes (DEGs) were detected to determine the similarities and differences among groups. We observed that many immune-related hallmark gene sets were significantly enriched in FA-treated groups (Groups F, FL, FM, and FMM), and these gene sets were then involved in an interferon-α response, an interferon-γ response, and TNFα signaling via NFκB. However, this significant enrichment cannot be observed in *S. aureus*-challenged groups (Groups L, M and MM) (Figure 3A and Figure S3), which may be explained by the immune evasion of *S. aureus*. GSEA results also showed that apoptosis in the hallmark gene set was enriched in the FA-treated groups (Figure S3). Furthermore, 12 concentration gradients of extra FA from 0.001 μg/mL to 20 μg/mL were applied to evaluate the cytotoxicity of FA itself to cells, and the results showed that only high concentration of FA with 10 μg/mL and 20 μg/mL induced cell death (Figure S2D). The Mac-T cells were treated with 0.001–5 μg/mL FA following bacterial treatment to investigate whether FA can influence the apoptosis induced by *S. aureus* or MRSA challenge. Considering the low cytotoxicity of the strain L of *S. aureus* (Figure 1D), strains M and MM with high toxicity were selected. The results showed that 0.001, 0.05, and 5 μg/mL FA reduced the apoptosis induced by strains M or MM of *S. aureus* at MOI 50. In particular, 0.05 μg/mL of FA was the most suitable concentration according to LDH cytotoxicity assay, mitochondrial membrane potential, and apoptosis detection (Figure 3B and Figure S4).

### 3.4. Transcriptional Changes of Mac-T Cells Induced by FA Supplementation and S. aureus Challenge

**Figure 4:**
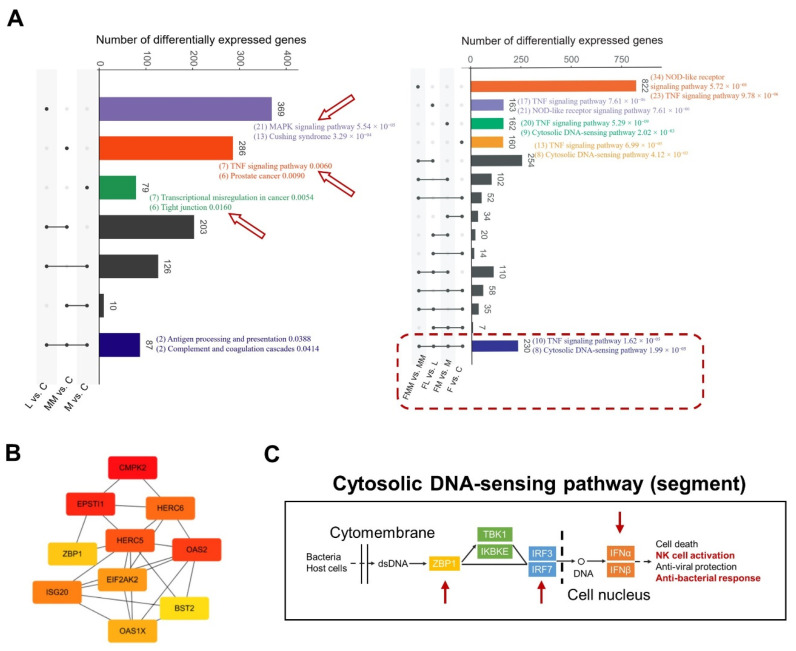
Cytosolic DNA-sensing pathway can be regarded as a key pathway for improving host anti-*S. aureus* and MRSA damage. (**A**) Enriched KEGG pathways and corresponding *p* values in common and unique differentially expressed genes among different comparison sets. (**B**) Top 10 hub genes of 230 common differentially expressed genes between FA treatment groups and corresponding controls. (**C**) Part of the genes involved in cytosolic DNA-sensing pathway, and DEGs in this pathway were marked by the red arrow.

DEGs were detected by the R packages of DEseq2 with the criteria of log_2_|Fold change| > 1 and *q* value < 0.05. We observed 785, 302, 586, 590, 834, 723, and 1663 DEGs in L vs. C, M vs. C, MM vs. C, F vs. C, FL vs. L, FM vs. M, and FMM vs. MM, respectively (Table S4). Only 87 DEGs were shared among the comparison sets between *S. aureus*/MRSA groups and control groups (left panel in Figure 4A), thus suggesting large differences in cellular responses induced by different *S. aureus* strains. A total of 230 DEGs were common among the comparison sets between FA treatment groups and corresponding groups without supplementation (right panel in Figure 4A).

The biological pathways represented by common and unique DEGs were then identified (Figure 4A and Figure S5A–D). Antigen processing and presentation and complement and coagulation cascades were enriched in the common DEGs, which thus might be used as stable markers to improve the resistance of mammary epithelial cells defense against *S. aureus*. By contrast, the unique genes induced by different *S. aureus* strains were enriched in the MAPK signaling pathway, TNF signaling pathway, and tight junction (*p* < 0.05, left in Figure 4A). Unlike the unique KEGG pathway enrichment results of DEGs among different *S. aureus*/MRSA-challenged and unchallenged groups, many DEGs between the FA-treated and untreated groups were significantly enriched in similar KEGG pathways (*p* < 0.01), such as NOD-like receptor signaling pathway, TNF signaling pathway, and cytosolic DNA-sensing pathway. This finding reflects the stable effects of FA on improving the host’s immune level prior to various types of *S. aureus*/MRSA challenge (right in Figure 4A).

### 3.5. FA Activates the Anti-Bacterial Responses by Elevating the Abilities of Cytosolic DNA Sensing and Tight Junction Assembly

The cytosolic DNA-sensing pathway, including *ZBP1*, was enriched by the overlapped genes between the common DEGs in FA-treated groups and the turquoise module of WGCNA (Figure S6). A hallmark gene set of interferon-α response at the downstream of cytosolic DNA-sensing pathway was enriched by GSVA/GSEA. The 10 hub genes, including *ZBP1*, identified with the common DEGs were closely related (Figure 4B). Thus, the *ZBP1* gene was selected for functional validation.

**Figure 5:**
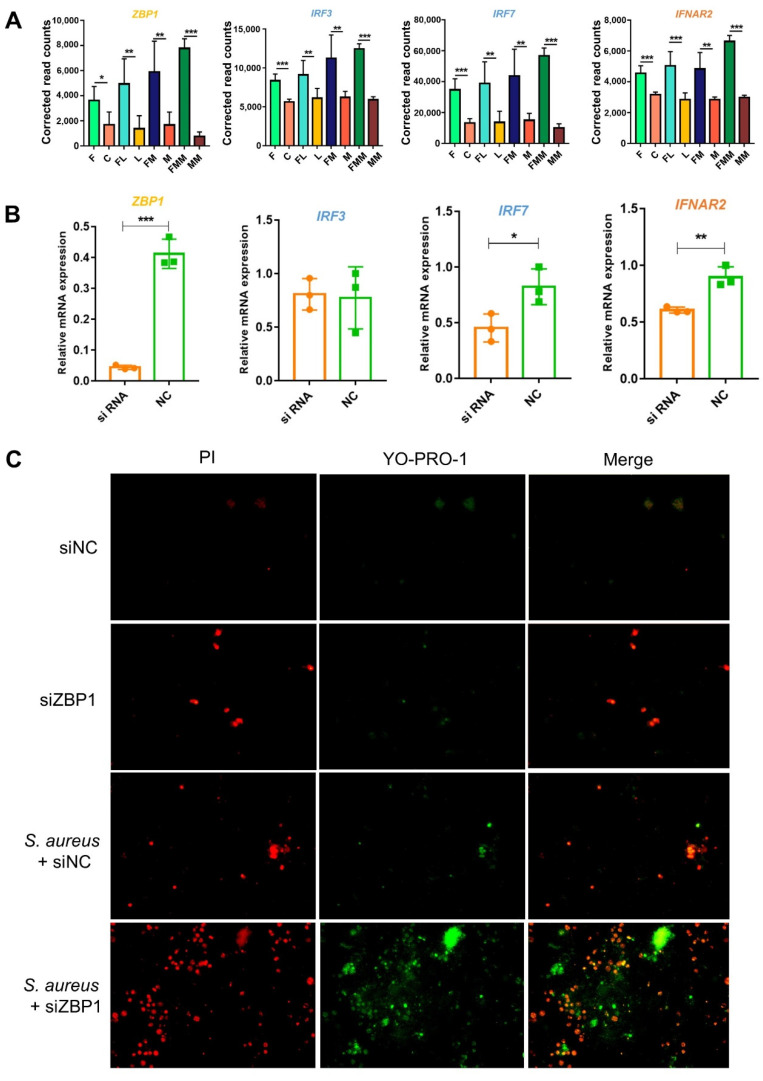
*ZBP1* can be regarded as a potential target of reducing the damage induced by *S. aureus* and MRSA in Mac-T. (**A**) In the FA treatment groups, the expression of *ZBP1*, *IRF3, IRF7,* and *IFNAR2* within the cytosolic DNA-sensing pathway is activated. (* *p* < 0.05, ** *p* < 0.01 and *** *p* < 0.001) (**B**) Relative mRNA expression of downstream genes of *ZBP1* after *ZBP1* knockdown. (**C**) The knockdown of *ZBP1* leads to cell apoptosis and necrosis. The nucleus of apoptotic cells were strained green by YO-PRO-1; The nucleus of necrotic cells were strained green and red by YO-PRO-1 and PI; the nucleus of normal cells cannot be stained any color. siNC: negative control.

In the cytosolic DNA-sensing pathway (Figure 4C), the upstream DEG *ZBP1*, the midstream *IRF3* (interferon regulatory factor 3), the *IRF7* (interferon regulatory factor 7), and the downstream *IFNAR2* (interferon alpha and beta receptor subunit 2) were significantly and highly expressed in FA-treated groups compared with those in the corresponding control groups (*p* < 0.05). By contrast, *S. aureus* challenge cannot effectively activate this pathway (Figure 5A and Figure S7A). Given that the cytosolic DNA-sensing pathway can activate a subsequent anti-bacterial response and *ZBP1* belongs to the top 10 hub genes, RNAi was conducted for *ZBP1*, and the efficiency was almost 90% (Figure 5B). Consistently, the expression of upstream *ZBP1*, midstream *IRF7*, and downstream *IFNAR2* were significantly lower in the siRNA group than in the NC group (Figure 5B, Figure S7B). Compared with the non-interference group, the interference of *ZBP1* leads to apoptosis and necrosis of cells, and this phenomenon was more serious when *S*. *aureus* challenged (Figure 5C). Therefore, in Mac-T cells, FA may elevate the perception of the host for bacteria by activating the expression of *ZBP1* within the cytosolic DNA-sensing pathway.

We observed that more percentage of exfoliated epithelial cells were detected in the MRSA-treated group (83.3%) compared with the control group (0%) (middle in Figure S8), while FA appears to reduce the adverse effect induced by MRSA (16.7%) (right in Figure S8). The genes expression and connection within the pathway of tight junctions were subsequently showed in Figures S9A,B and S10A,B. The expression trend of the downstream DEGs of this pathway was consistent with the phenotype of the epithelial cells, thus highlighting the potential mechanism of FA in aiding mammary epithelial cells defense against the damage caused by *S. aureus* and MRSA.

### 3.6. Differentially Spliced Transcripts Are Enriched in Immune or Inflammatory Pathways

DEGs and differentially spliced (DS) transcripts participate in host immune or inflammatory responses. Leafcutter was used to examine DS events between FA-treated and *S. aureus*-challenged groups and corresponding control groups with the criteria of *q* < 0.05. In the comparison between *S. aureus*-challenged and unchallenged groups, 111, 19, and 113 differentially spliced genes (DSGs) were identified between L and C, M and C, MM and C, respectively (Table S4). In the comparison between FA-treated groups and the controls, 116, 86, 108, and 275 DSGs were identified between F and C, FL and L, FM and M, and FMM and MM, respectively (Table S4).

**Figure 6:**
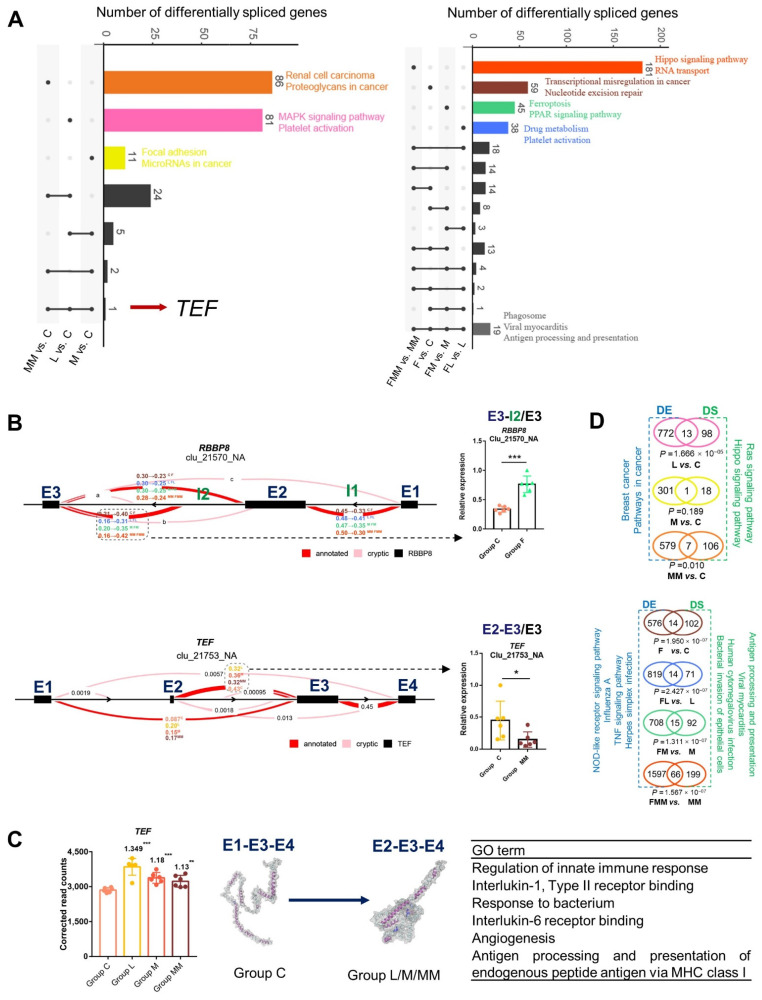
Differentially spliced genes and their relationship with DEGs. (**A**) Enriched KEGG pathways in common and unique differentially spliced genes among different comparison sets. (**B**) Alternative splicing types of the two common DS genes (*RBBP8* and *TEF*) (left panel) and the corresponding expression levels of the part in the grey dotted box was verified by qRT-PCR (left panel). E: exon; I: intron. (**C**) Transcriptional expression of *TEF* in the four groups (left panel), predicted protein conformation of the two types of *TEF* DSs using I-TASSER (middle panel) and protein conformation of E2-E3-E4-related GO terms (right panel). (**D**) Relationship between DE and DS genes. Overlaps were tested by the hypergeometric test. The left and right sides are the respective pathways using DEGs and DSGs, respectively. * *p* < 0.01, ** *p* < 0.01, *** *p* < 0.001.

Similar to the KEGG enrichment results of DEGs, the DSGs were mainly enriched in immune-related pathways, such as antigen processing and presentation, MAPK signaling pathway, and PPAR signaling pathway (Figure 6A and Figure S11A–D). Similar to the aforementioned few common DEGs, only one and 19 DSGs were common among the three *S. aureus* strains-challenged groups and FA treatments compared with their corresponding controls, respectively (Figure 6A,B).

Two common DS genes, *RBBP8* and *TEF*, were chosen to validate the reliability of Leafcutter analysis (left in Figure 6B) by qRT-PCR (right in Figure 6B). Good consistency between both assays was achieved. In addition, the protein conformation of the common DSG *TEF* (TEF transcription factor) in *S. aureus*-treated groups was analyzed by I-TASSER (middle in Figure 6C). Compared with the E1-E3-E4 protein conformation corresponding to the main DS type in controls, more immune response-related GO terms were related to the protein conformation of E2-E3-E4 corresponding main DS type in *S. aureus*-treated groups (right in Figure 6C). *TEF* expression was also higher in *S. aureus*-treated groups than in controls (left in Figure 6C). Moreover, significant correlations were found between *TEF* and its target genes *MYCN* and *LEF1* (Figure S12). In conclusion, *TEF* can be regarded as a critical biomarker gene related to immune responses of bovine mammary epithelial cells to *S. aureus* or MRSA challenge.

The DSGs and DEGs were compared for the seven sets (L vs. C, M vs. C, MM vs. C; F vs. C, FL vs. L, FM vs. M, and FMM vs. MM). Although the number of the overlapped genes in each comparison set was small, the intersection was significantly higher than expected (hypergeometric *p* value < 0.05) (Figure 6D). The DSGs and DEGs were enriched in distinct immune-related KEGG pathways (Figure 6D, Figures S5 and S11). These findings imply that the host can widely mobilize either DS events or DE changes to induce immune or inflammatory responses.

### 3.7. Association of Key Modules and Gene Sets with Complex Traits

**Figure 7:**
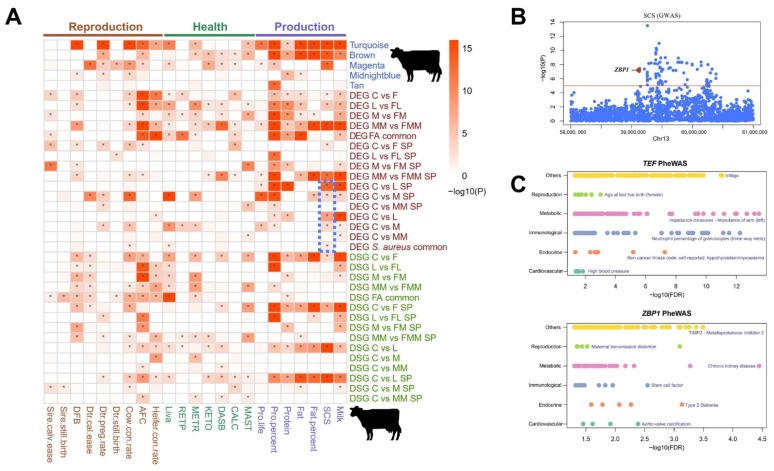
Characterization of GWAS signal enrichment and Phe-WAS. (**A**) GWAS enrichment analysis in bovine large-scale GWAS data (* *p* < 0.05). SP: specific DEGs or DSGs in a comparison set; MAST: mastitis; CALC: Hypocalcemia; DASB: Displaced abomasum; KETO: Ketosis; METR: Metritis; RETP: Retained placenta; Liva: Livability. (**B**) Significant GWAS signal of bovine SCS trait found around bovine *ZBP1*. (**C**) Phe-WAS analyses of the important candidate genes (*ZBP1* and *TEF*) in human large-scale phenotypic data.

To investigate the association of modules or gene sets with the GWAS signals of 23 bovine reproduction, health, and production traits, we selected 5 modules from WGCNA and 16 gene sets from DEGs as well as 15 gene sets from DSGs to perform GWAS signal enrichment analysis (Table S5). As shown in Figure 7A, turquoise, brown, and magenta modules are enriched in several GWAS signals including SCS and mastitis (MAST) (*p* < 0.05). Common DEGs and DSGs in FA-treated groups are widely enriched in many reproduction, health, and production traits, implying that the multiple effects of FA need to be further explored. Common DEGs in *S. aureus*-infected groups were significantly related with SCS, livability, and cow conception rate traits (Figure 7A, Table S6). Significant enrichment with SCS trait was observed for the DEGs between *S. aureus*-challenged and unchallenged groups (C vs. L, C vs. M, and DEGs *S. aureus* common) but not between MRSA-challenged groups and controls (DEG C vs. MM and DEG C vs. MM SP) (*p* < 0.05). A possible reason is the low incidence of MRSA infection and the rarity of related GWAS signals in dairy cattle herds. Given the strong antibiotic resistance of MRSA, attention needs to be directed to the specific DEGs between C and MM.

Compared with those in previous large-scale bovine GWAS data, significant SNPs existed for the critical candidate genes of *ZBP1* (Figure 7B) and for *ROCK2*, *SCRIB,* and *ERBB2* within the pathway of tight junctions (Figure S12A). These findings verified the reliability of this research and the genes as targets for enhancing bovine resistance to *S. aureus* mastitis (Figure 7B and Figure S13A).

Phenome-wide association analysis (Phe-WAS) were conducted for human complex traits to explore whether the key genes about bovine mammary epithelial cell inflammation and immune responses are functionally similar in humans. Phe-WAS results revealed that key genes, such as DEGs *ZBP1*, genes within the tight junction pathway, and DSG *TEF*, were significantly associated with several human immunological traits (Figure 7C, Figure S13B, Table S7). These findings suggest the conserved function of these genes across species and their potential benefits for improving human health-related traits.

## 4. Discussion

With the growing antibiotic resistance of pathogens, investigations on host–microbe interactions have become critical. Livestock-associated (LA) antibiotic-resistant strains of *S. aureus*, such as MRSA, can be transmitted from animals to humans [29,30,31]. Therefore, the immune process of bovine mastitis induced by *S. aureus*, especially MRSA, and the enhancing effect of micronutrient supplementation on animal disease resistance needs to be comprehensively analyzed. This study described the transcriptomics of the immune responses of Mac-T cells with or without FA treatment to different *S. aureus* strains challenge, and revealed the key WGCNA modules, DEGs, DSGs, and corresponding pathways that are beneficial for the prevention and treatment of zoonotic pathogens. Results can be summarized in three findings.

First, the low common DEGs and DSGs induced by different *S. aureus* lineages showed the great heterogeneity of host immune responses to different *S. aureus* lineages. The challenge of *S. aureus* strains with different lineages in bovine mammary epithelial cells results in the differential expression of genes related to inflammation [9,32]. Among which, only a few candidate genes were tested by low-throughput qPCR or ELISA. However, bacterial infection usually changes the expression of a number of genes [33]. Therefore, this study comprehensively analyzed the transcriptomic characteristics of Mac-T cells challenged by three different *S. aureus* strains from the aspects of gene expression and gene alternative splicing. DEGs and DSGs (L/M/MM vs. C) show that different *S. aureus* strains can activate moderately common DEGs and DSGs, but induces numbers of strain-specific DEGs and DSGs. These genes are mostly enriched in inflammation and cancer-related pathways [34,35]. *S. aureus* DEGs are commonly involved in immune pathways, such as antigen processing, presentation, complement, and coagulation cascades (Figure 4A) [36,37]. Furthermore, the unique common DSGs in *S. aureus*-challenged groups (*TEF*, left in Figure 6A) are related to the progress of cellular death and cancer [38,39,40]. These genes provide further broad-spectrum data for anti-*S. aureus* mastitis strategies. In addition, MRSA-specific DEGs and DSGs could provide the answer for highly effective prevention strategies for the strong antibiotic-resistant strain MRSA induced by antibiotics. Although the overlapped genes between DEGs and DSGs are significant (Figure 6D), the inflammatory pathways regulated by these genes are almost different. This finding was similar with a previous study [41] and highlighted the host’s own comprehensive response abilities to defend against *S. aureus* [42]. In the present study, one-time point comparison was adopted to explore the interplays between Mac-T cells and *S. aureus*. Because the changes in gene expression with the duration of bacterial infection [43,44], further studies about the time gradients of *S. aureus* infection need to be conducted.

Second, 5 μg/mL of FA can significantly improve the abilities of Mac-T cells defense against MRSA treatment by enhancing cytosolic DNA sensing and tight junction assembly. Mammary epithelial cells play a prominent role in the recognition of pathogens and the initiation of innate immune cells [45]. However, the influence of FA supplementation on the anti-bacterial abilities of mammary epithelial cells is largely unknown. Here, high FA doses were found to promote apoptosis of Mac-T cells, but 5 μg/mL of FA can reduce the apoptosis of the cells induced by *S. aureus* challenge. This finding is in agreement with previous studies stating that FA has a “double-edge sword” effect [18,22,46,47,48,49,50,51]. Thus, the benefits or downsides of FA for cell apoptosis need to be considered in its future application.

To the best of the authors’ knowledge, this work is the first to reveal the comprehensive effects of FA on enhancing the abilities of Mac-T cells defense against *S. aureus* damages. On the one hand, effective detection and response to causative agents are essential functions of the innate immune systems [52]. The results showed that the obviously activated ZBP1, IRF3, IRF7, and IFNAR2 within cytosolic DNA-sensing pathway was enriched in the FA-treated groups but not in the *S. aureus* challenge groups (Figure 5A). As a DNA sensor, *ZBP1* within the upstream of this pathway is involved in anti-pathogen mechanism and inflammation mediation [53,54,55]. Significant SNPs associated with SCS were observed around bovine *ZBP1* through GWAS enrichment analysis (red dots in Figure 7B), which supported the results of in vitro. Thus, the protective function of *ZBP1* against mastitis needs to be considered. On the other hand, the tight junction pathway also plays a vital function in the normal epithelial barrier protect against pathogens [56]. *S. aureus* and MRSA disrupted the tight junction and suppressed its assembly [57,58,59,60], and FA was able to reduce the damage of *S. aureus* and MRSA to Mac-T cells’ tight junction (Figures S8 and S9). The genes of *SCRIB*, *ERRB2,* and *ROCK2* within tight junction pathway were also found to be associated with the GWAS signals of bovine SCS trait (Figure S13). Thus, the effect of FA on cytosolic DNA sensing and tight junction pathways provides a new insight into the prevention of diseases induced by *S. aureus* and its MRSA.

Third, the results provide information to prevent mammary inflammatory-related diseases. It is worth pointing out that we just used Mac-T cells to investigate the influence of *S. aureus* challenge and FA treatment on host immune responses. Mac-T is one of the bovine mammary epithelial cell lines. It was produced from primary bovine mammary alveolar cells by stably transfected with SV40 large T-antigen [61]. On the one hand, Mac-T cells can normally secrete casein proteins [61]; on the other hand, they can properly reflect main features of pathogen species-specific immune response of the parental cell [62]. However, the bovine mammary gland does not only consist mammary epithelial cells, it is a complex organ consisted of various cell types [63]. Therefore, the results produced from the Mac-T cell model were further confirmed by using cattle large-scale GWAS data in the current study. Through GWAS enrichment analysis and Phe-WAS, key genes, DEG *ZBP1* and DSG *TEF*, were verified, which can be regarded as promising targets for improving bovine *S. aureus* mastitis defense and public health.

In conclusion, our study elucidates the cross-talk between bovine Mac-T cells, *S*. *aureus*/MRSA strains, and FA by combining molecular phenotypes of transcriptomes with functional validation. We reveal a strain-specific immune response of Mac-T cells to different *S. aureus* strains on a genome-wide transcriptional level. At the same time, the common DEGs in the Mac-T cells induced by the three *S. aureus* stains could be considered as stable indicators of bovine mammary epithelial cells response to the challenge of *S. aureus*. Our work indicate that FA could help Mac-T cells defense against *S. aureus* and MRSA challenge through activating the pathway of cytoplasmic DNA sensing. Knockdown of *ZBP1*, an upstream gene in this pathway (Figure S14), had a significant effect on the responses of host cells induced by *S. aureus* and MRSA.

## Figures and Tables

**Table 1:** Primer information used in this study.

Gene Names	Forward (5′-3′)	Reverse (5′-3′)
*Nuc*	GCGATTGATGGTGATACGGTT	AGCCAAGCCTTGACGAACTAAAGC
*MecA*	GTAGAAATGACTGAACGTCCGATAA	CCAATTCCACATTGTTTCGGTCTAA
*TEF-AS*	GGTGGCTGAGCTAGAAGGG	CAGGGTCGGGATTGAAGT
*TEF*	GCCTCCGAACAGACAAATC	AATCAAGGGTCACTGCTACG
*RBBP8-AS*	GTTAGTCAGGGAACGAGG	AGGGCTTCCACAACTGCT
*RBBP8*	AGCCAAGGATGTGAGA	TGGACGAAGAGGGATT
*ZBP1*	CAGGAGACACAGACCTTGAGCAG	CATCTTGTGGAGGAGCTGGTT
*GAPDH*	GGTGCTGAGTATGTGGTGGA	GGCATTGCTGACAATCTTGA
*IFNAR1*	TCTGCGTCCTTTGCC	CACAGGGCTGCTTACA
*IFNAR2*	CTGTGTGTGTGAGAGCCCTT	GGCCACCAAAACGCTTGATT
*IRF3*	CGACCCAACACTTAGGCCAG	GTCGGGCTTATCCTTCCCAG
*IRF7*	GACTTCGGCACCTTCTTCCA	TAGATGGTGTAGTGCGGGGA
*TBK1*	GGATGAGGGACAATTCTGTGTCT	ACCAAAACCTAATCCTTCTGGG
*IKBKE*	GGCCTCTCTGCTCAACACAT	ATCTCCACGAACCAGTGCAG

## Data Availability

Raw transcriptomic data were deposited in NCBI (PRJNA723165), *S. aureus* resequencing data were deposited in NCBI (PRJNA723407). All relevant data supporting the conclusions of this study are included in the manuscript and the Supplementary Files. Requests to access any other data should be directed to the corresponding author: Ying Yu at yuying@cau.edu.cn.

## Supplementary Materials

The following are available online at https://www.mdpi.com/article/10.3390/biology10111164/s1, Figure S1: Phylogenetic tree construction of dairy cow milk *S. aureus* whole genome sequences. Figure S2: The interaction between folic acid, *S. aureus* and Mac-T cells. Figure S3: Thermogram of interferon α response and apoptosis in FA treatment groups by GSEA analysis. Figure S4: Influence of different doses of FA on apoptosis (tested by LDH release assay) induced by *S. aureus* strains M and MM. Figure S5: DEGs enriched GO terms and KEGG pathways. Figure S6: Conjoint analysis of common-DEGs and WGCNA modules. Figure S7: mRNA expression of downstream genes of *ZBP1*. Figure S8: Observation of exfoliated epithelial cells in MRSA and FA-treated epithelial cells (six samples/group). Figure S9: Expression heatmaps of some genes of the tight junction pathway. Figure S10: Gene expression changes of tight junction pathway in MM vs. C (A) and FMM vs. MM (B). Figure S11: DSGs enriched GO terms and KEGG pathways. Figure S12: Regression between *TEF* and its target genes *MYCN* and *LEF1*. Figure S13: GWAS enrichment and Phe-WAS analysis. Figure S14: Summary of the effects of folic acid and MRSA on Mac -T cells. Table S1: Reads summary. Table S2: WGCNA modules. Table S3: DEGs summary. Table S4: DEGs and DSGs details. Table S5: Modules and gene sets used for GWAS enrichment. Table S6: GWAS enrichment. Table S7: PheWAS summary.

## Abbreviations

AFC	Age at first calving
pbMECs	Primary bovine mammary epithelial cells
BMI	Body mass index
CFU	Colony-forming unit
CALC	Hypocalcemia
Cow.con.rate	Cow conception rate
DASB	Displaced abomasum
DEG	Differentially expressed gene
DFB	Days to first breeding
DMEM	Dulbecco’s modified Eagle’s medium
DSG	Differentially spliced gene
Dtr.cal.ease	Daughter calving ease
Dtr.preg.rate	Daughter pregnancy rate
Dtr.still.birth	Daughter stillbirth
FA	Folic acid
Fat	Fat yield
GO	Gene Ontology
GSEA	Gene Set Enrichment Analysis
GSVA	Gene Set variation Analysis
GWAS	Genome-wide association studies
Heifer.con.rate	Heifer conception rate
KEGG	Kyoto Encyclopedia of Genes and Genomes
KETO	Ketosis
LDH	Lactate dehydrogenase
Liva	Livability
Mac-T	Bovine mammary alveolar cells
MAST	Mastitis
METR	Metritis
Milk	Milk yield
MLST	Multilocus sequence typing
MOI	Multiplicity of infection
MRSA	Methicillin-resistant *S. aureus*
OD	Optical density
PCA	Plate count agar
PCA	Principal component analysis
PCR	Polymerase chain reaction
Phe-WAS	Phenome-wide association study
Pro.life	Productive life
Pro.percent	Protein percentage
Protein	Protein yield
RETP	Retained placenta
RIN	RNA integrity number
RT	Reaction time
*S. aureus*	*Staphylococcus aureus*
SCC	Somatic cell count
SCS	Somatic cell score
Sire.calv.ease	Sire calving ease
Sire.still.ease	Sire stillbirth
*TEF*	TEF transcription factor, PAR bZIP family member
WGCNA	Weighted Correlation Network Analysis
*ZBP1*	Z-DNA binding protein 1
