# Supplementary material for: Protective Roles of Folic Acid in the Responses of Bovine Mammary Epithelial Cells to Different Virulent Staphylococcus aureus Strains
Source: Biology (Basel). 2021 Nov 12;10(11):1164. doi: 10.3390/biology10111164 (PMC8615268; doi:10.3390/biology10111164)
Supplement: Supplementary file 1 [file biology-10-01164-s001.zip › Supplmentary figures (biology-1254790).pdf]

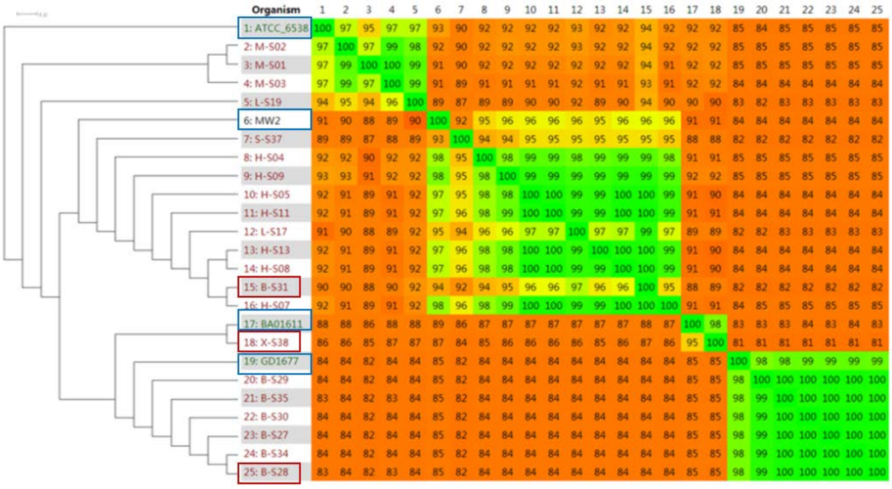


**Figure S1. Phylogenetic tree construction of dairy cow milk *S. aureus* whole genome sequences.** The red boxes represent the three *S. aureus* strains chosen for this study, and the blue boxes represent reference strains. B-S28: *S. aureus* isolated from cow with low milk somatic cell counts (MLST: ST398, abbreviated as L); B-S31: *S. aureus* isolated from cow with mastitis (MLST: ST1, abbreviated as M); B-S38: MRSA isolated from cow with mastitis (MLST: ST9, abbreviated as MM).

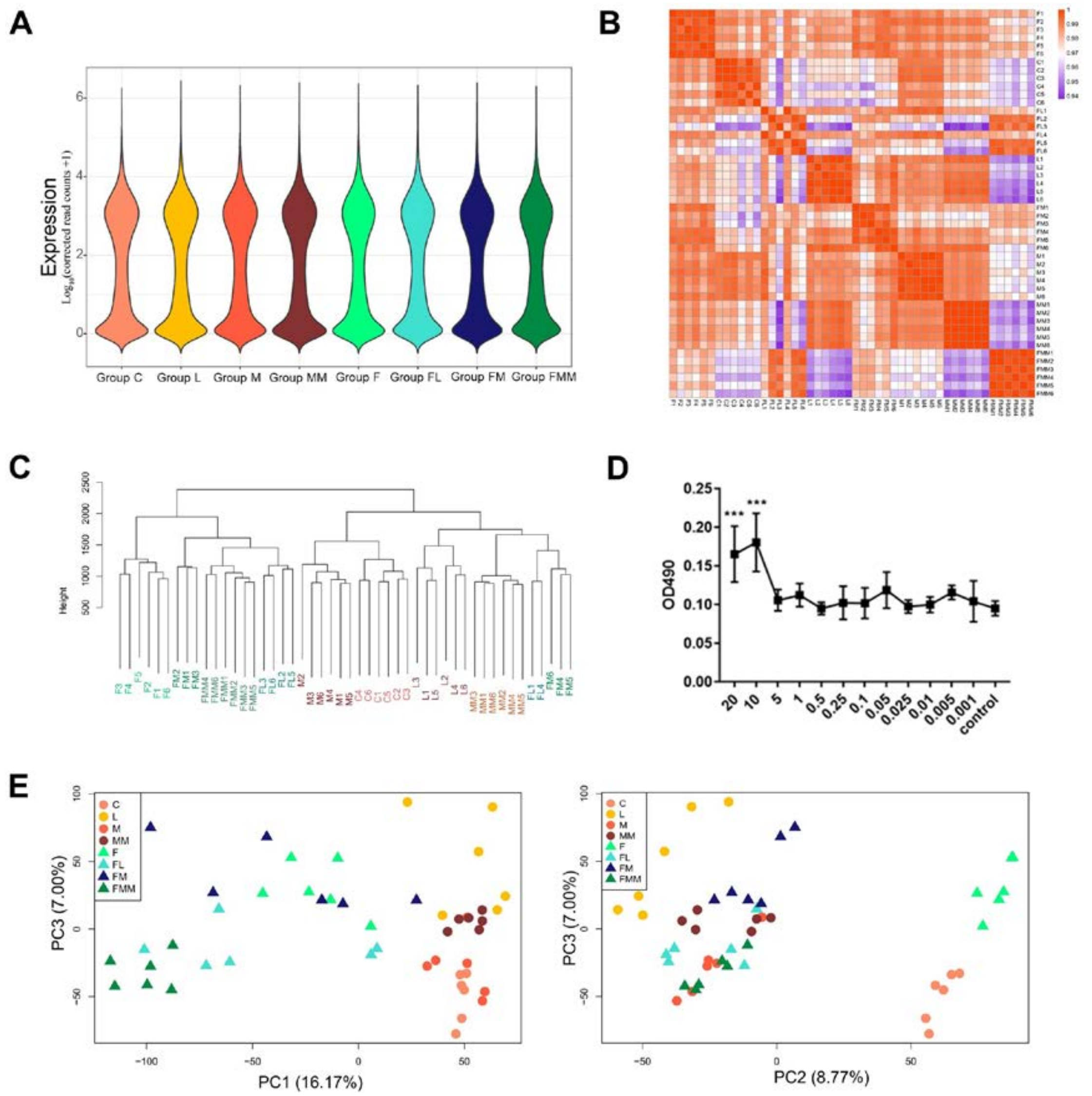


**Figure S2. The interaction between folic acid, *S. aureus* and Mac-T cells.** (A) Gene expression distribution among eight groups. (B) Similarities among 48 samples from eight groups in RNA-seq. (C) Cluster dendrogram of all samples. (D) The cytotoxicity of FA itself to Mac-T cells. (E) PCA analysis of all samples.

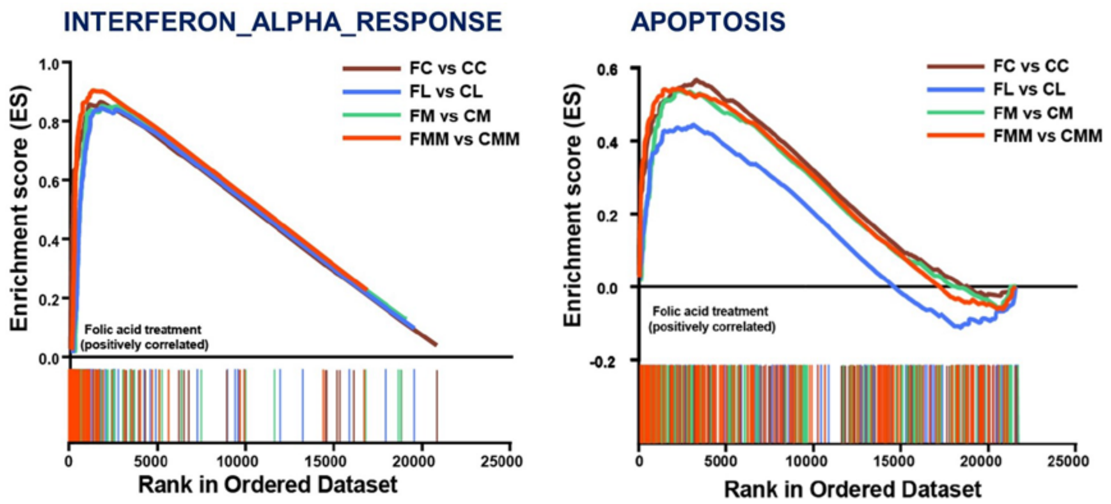


**Figure S3. Thermogram of interferon α response and apoptosis in FA treatment groups by GSEA analysis.**

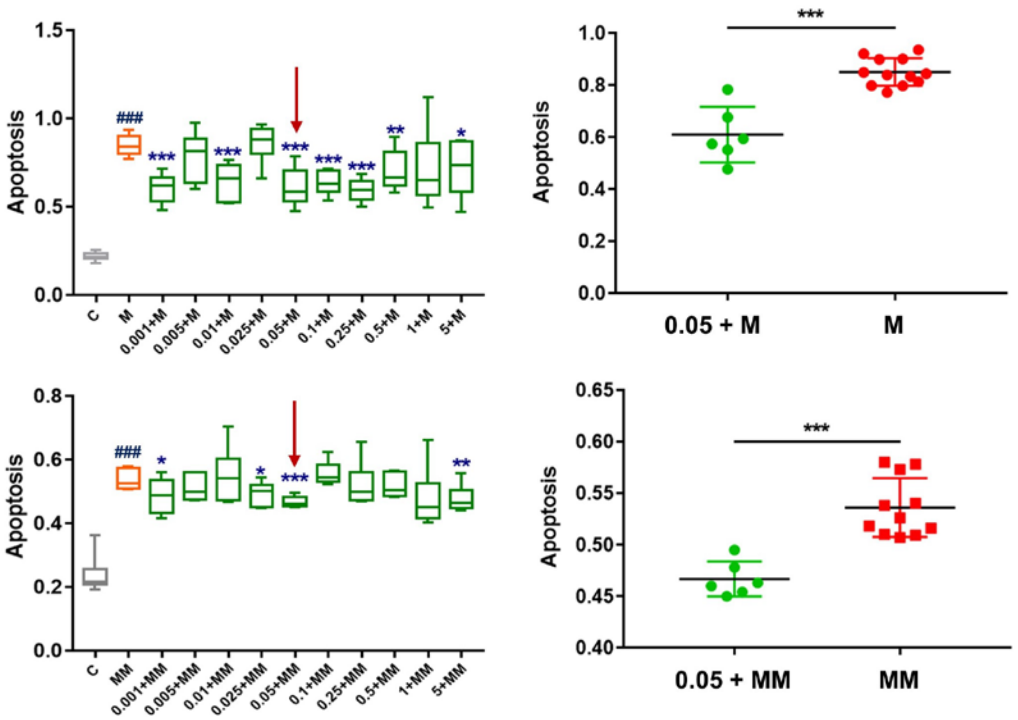


**Figure S4. Influence of different doses of FA on apoptosis (tested by LDH release assay) induced by *S. aureus* strains M and MM**. # shows the comparisons between the control cells and the cells treated with *S. aureus* infection, and * shows the comparisons between the cells treated with *S. aureus* infection and the cells treated with FA supplementation following with *S. aureus* infection.

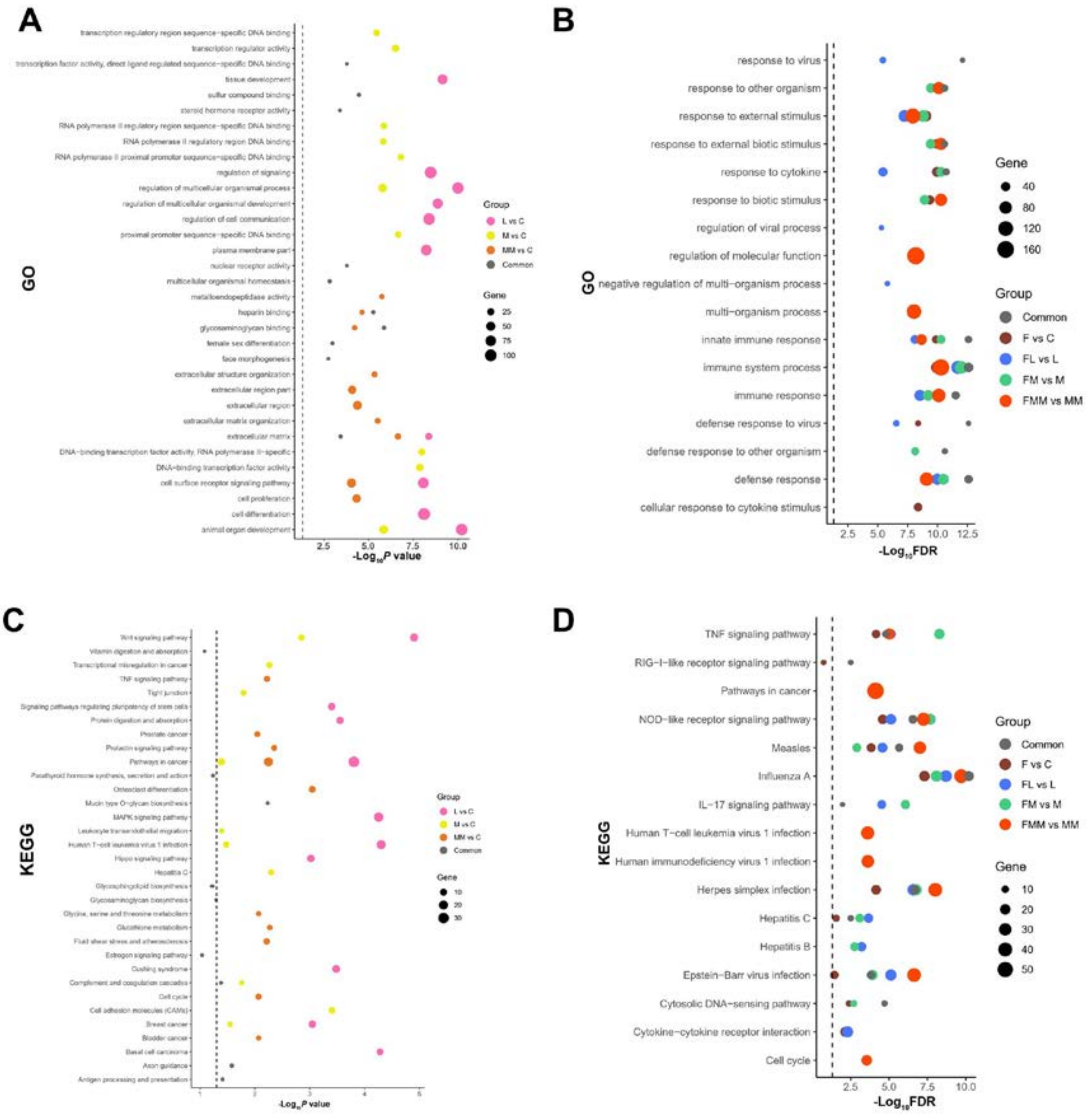


**Figure S5. DEGs enriched GO terms and KEGG pathways.** Enriched GO terms of DEGs from *S. aureus* infection (A) and folic acid treatment (B). Enriched KEGG pathways of DEGs from *S. aureus* infection (C) and folic acid treatment (D). Common: Common-DEGs among different comparisons.

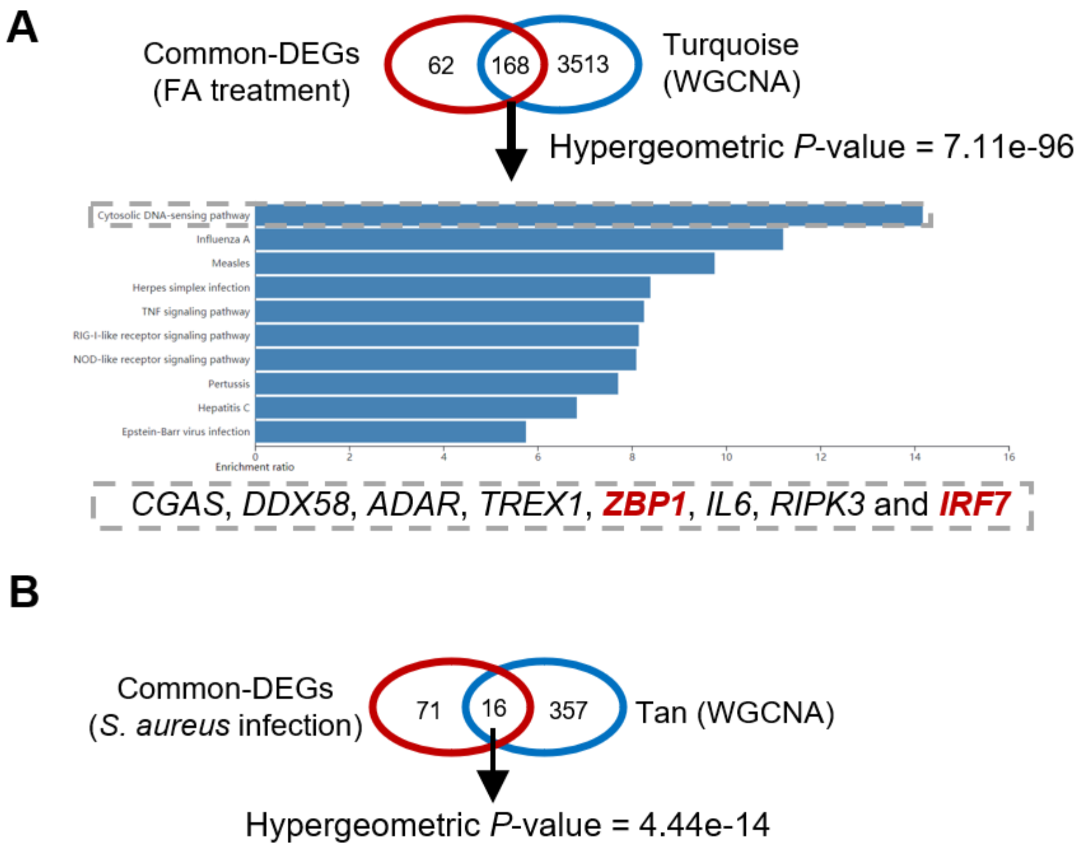


**Figure S6. Conjoint analysis of common-DEGs and WGCNA modules.** (A) The overlap between common-DEGs of FA treatment and WGCNA module Turquoise. (B) The overlap between common-DEGs of *S. aureus* infection and WGCNA module Tan.

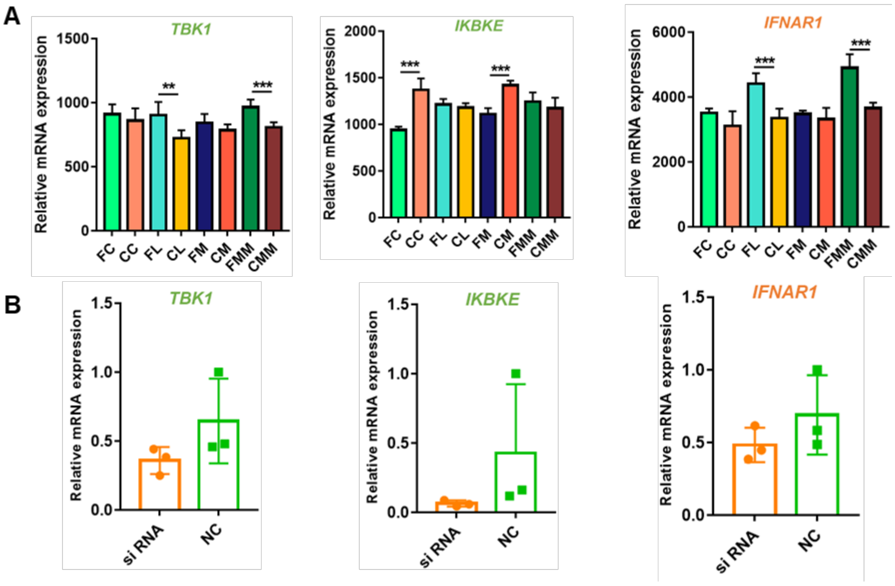


**Figure S7. mRNA expression of downstream genes of *ZBP1*.** (A) The mRNA expression of *ZBP1* downstream genes among eight groups. (B)After *ZBP1* knockdown, the relative mRNA expression of downstream genes of *ZBP1*.

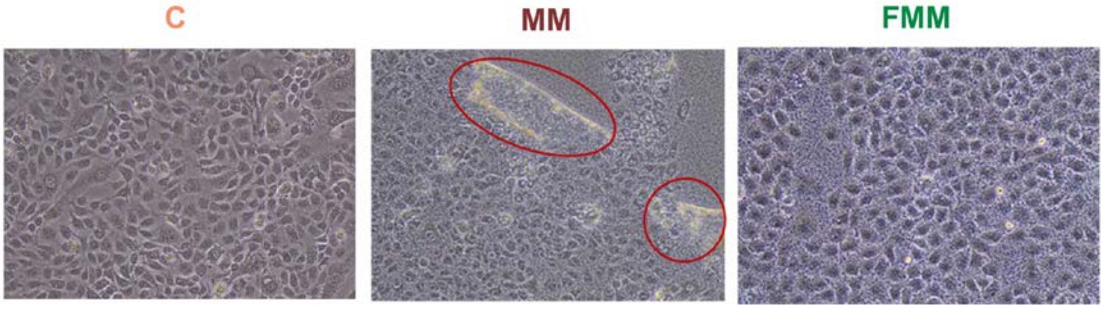


**Figure S8. Observation of exfoliated epithelial cells in MRSA and FA-treated epithelial cells (six samples/group).**

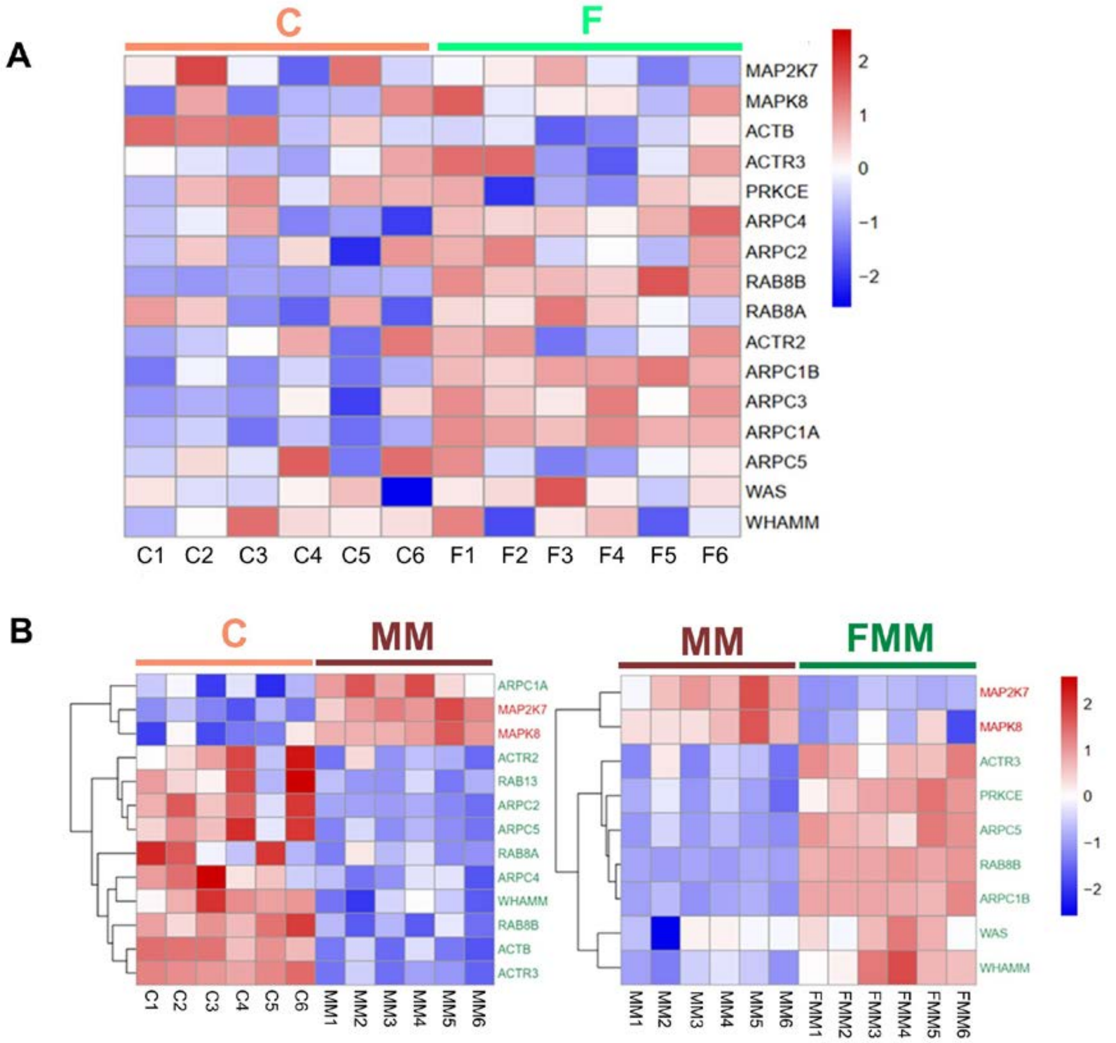


**Figure S9. Expression heatmaps of some genes of the tight junction pathway.** (A) Groups C and F. (B) Groups C and MM; Groups MM and FMM.

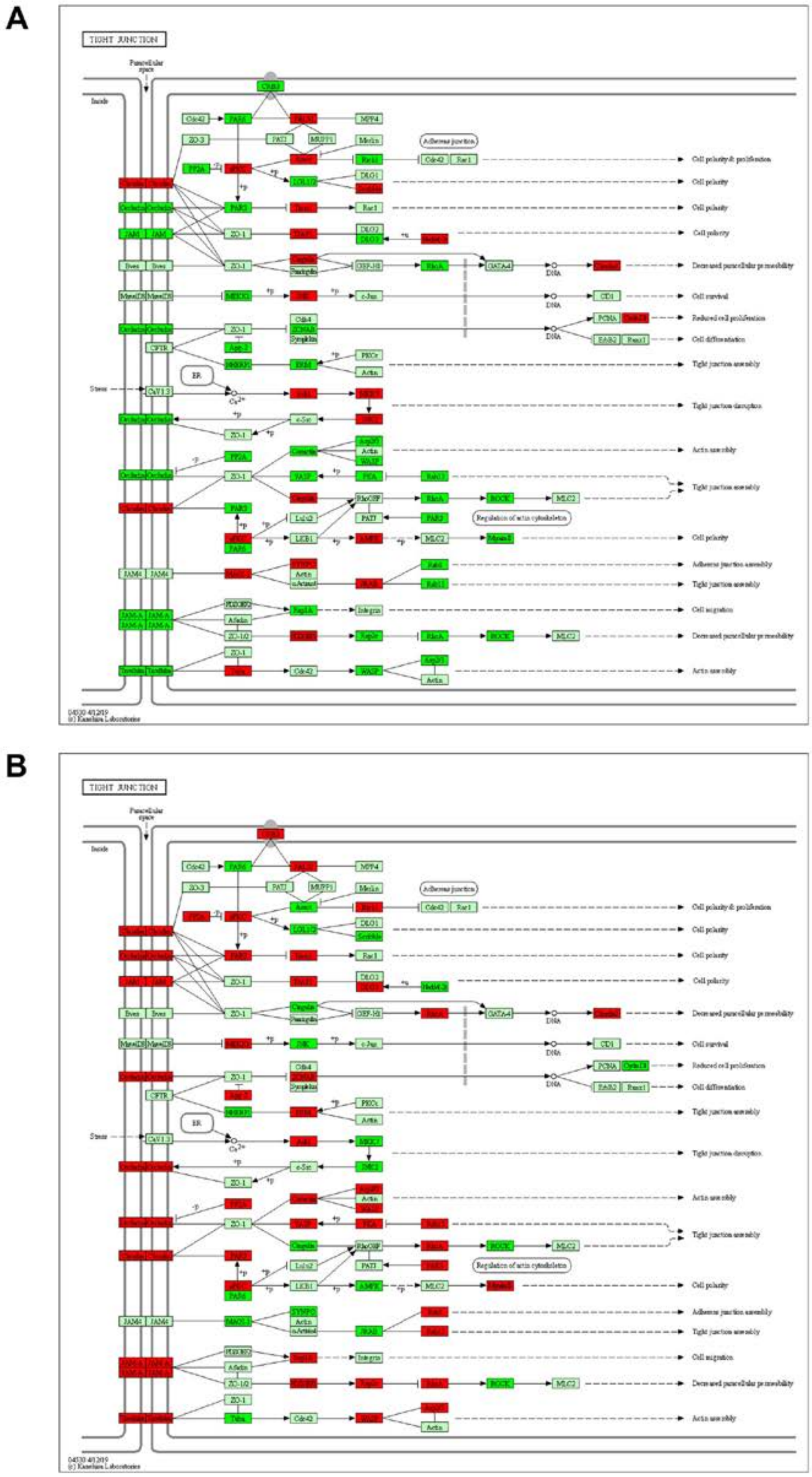


**Figure S10. Gene expression changes of Tight junction pathway in MM *vs.* C (A) and FMM *vs.* MM (B).** The red shading gene means expression up-regulation, and the green shading gene means expression down-regulation.

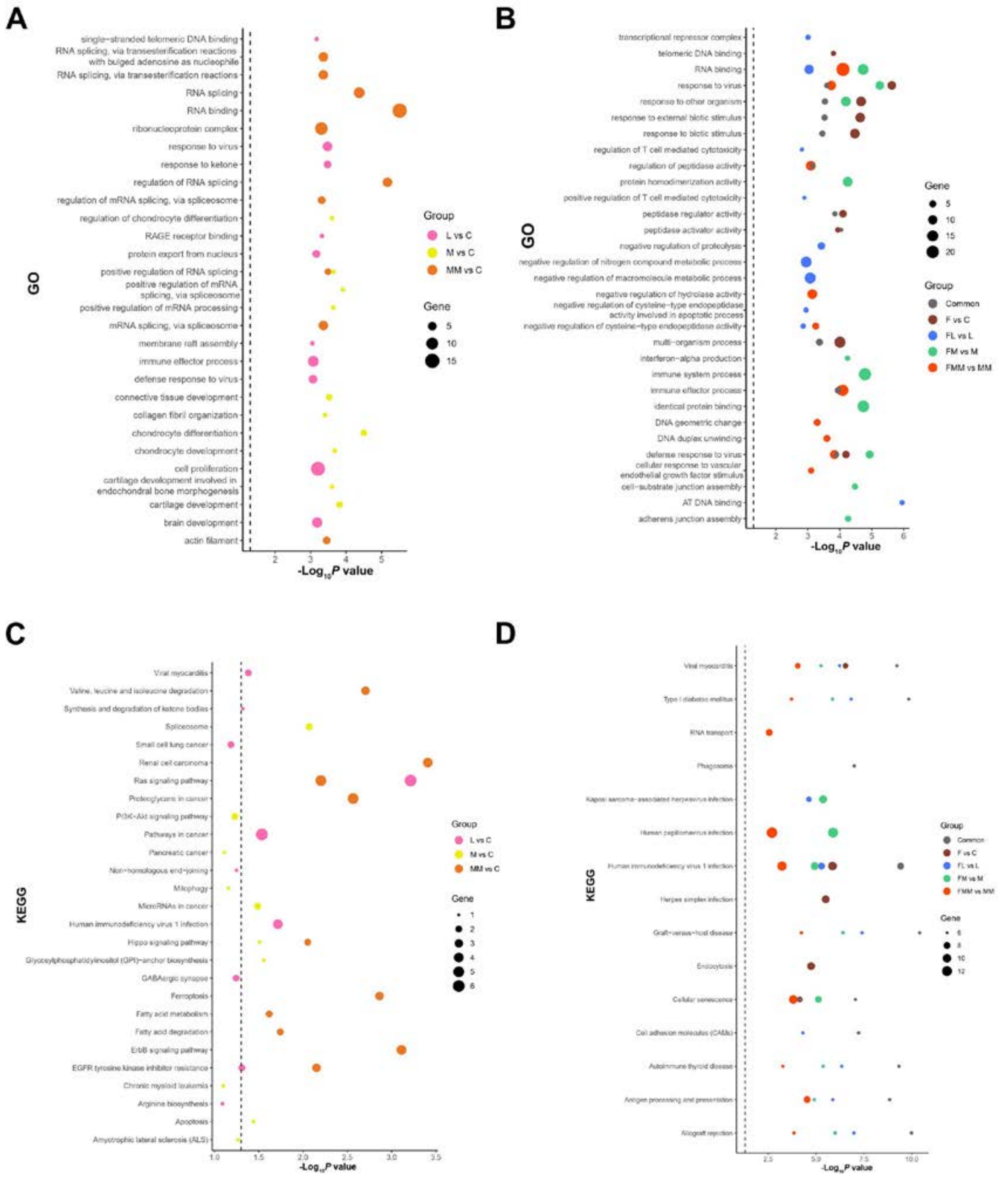


**Figure S11. DSGs enriched GO terms and KEGG pathways.** Enriched GO terms of DSGs from *S. aureus* infection (A) and folic acid treatment (B). Enriched KEGG pathways of DSGs from *S. aureus* infection (C) and folic acid treatment (D). Common: Common-DSGs among different comparisons.

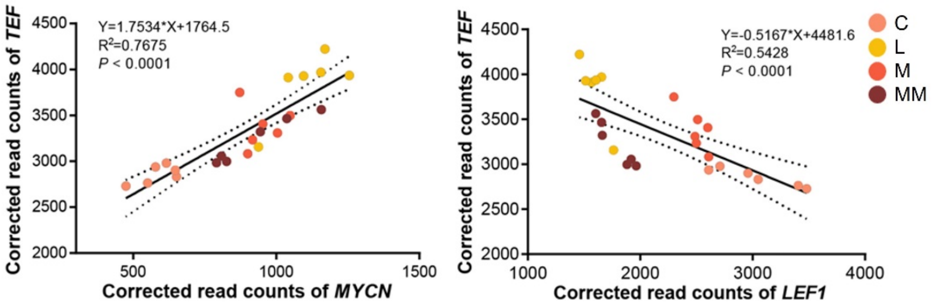


**Figure S12. Regression between *TEF* and its target genes *MYCN* and *LEF1*.**

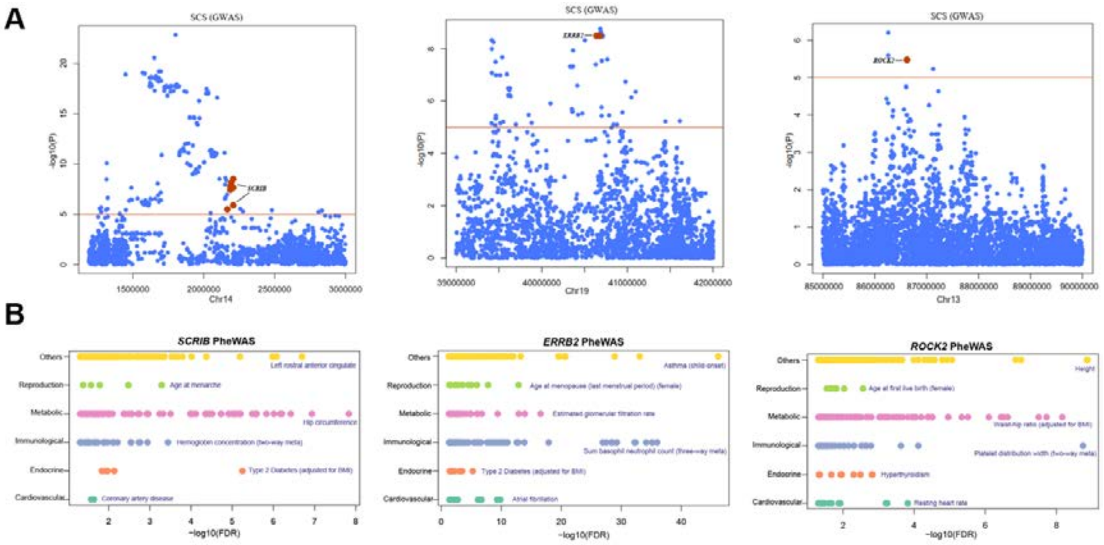


**Figure S13. GWAS enrichement and Phe-WAS analysis.** (A) Significant GWAS signals of bovine SCS trait can be found around bovine *SCRIB*, *ERRB2* and *ROCK2*. (B) Phe-WAS analyses of the important candidate genes (bovine *SCRIB*, *ERRB2* and *ROCK2*) in human large-scale phenotypic data.

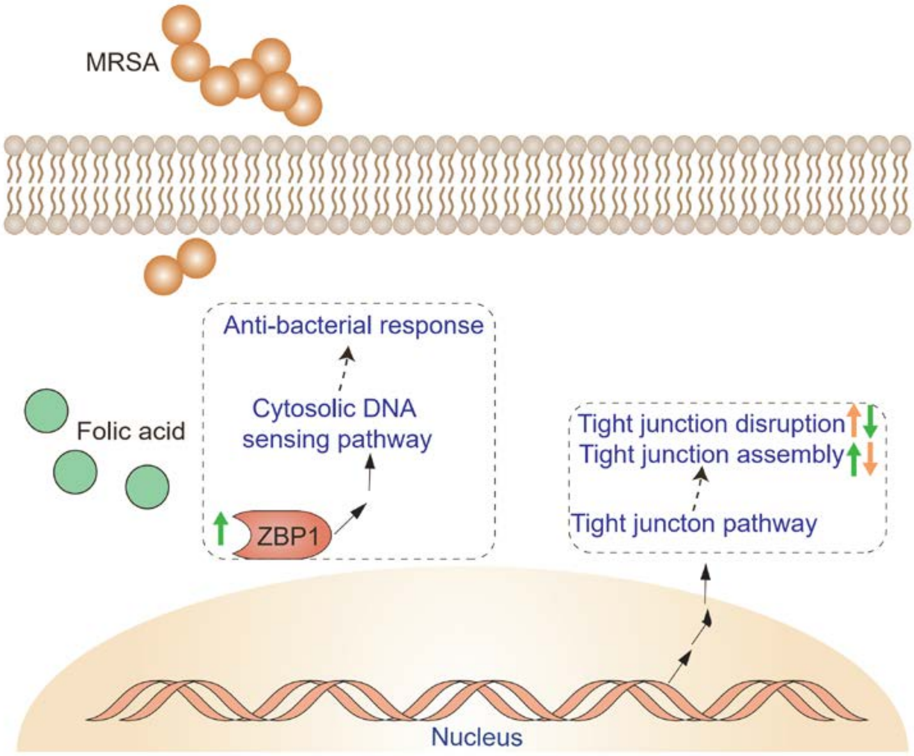


**Figure S14. Summary of the effects of folic acid and MRSA on Mac -T cells.** The orange and green arrows represent the rise and fall of gene expression, respectively.
